# 

**Published:** 1899-09

**Authors:** 


*The following donations have been received since the publication of the list in June:*

*August 31st, 1899.*

| | |
|---|---|
| L. M. Griffiths (1) ... ... ... ... ... ... ... | 56 volumes. |
| F. E. Hare, M.D. ... ... ... ... ... ... ... | 1 volume. |
| Official Board of Advertising for the Isle of Man (2) | 1 ,, |
| Medical Faculty, McGill University (3) ... ... | 1 ,, |
| Norske Medicinske Selskabs Bibliotek (4) ... ... | 35 volumes. |
| Editor of *The Railway Surgeon* (5) ... ... ... ... | 1 volume. |
| Royal Medical and Chirurgical Society (6) ... ... | 5 volumes. |
| R. Shingleton Smith, M.D. (7) ... ... ... ... | 1 volume. |
| Editor of *Virginia Medical Semi-Monthly* (8) ... ... | 1 ,, |
| Cyril H. Walker, M.B. (9) ... ... ... ... ... | 1 ,, |

Unbound periodicals and a framed picture have been received from Mr. L. M. Griffiths. Unframed pictures have been presented by Dr. J. O. Symes and Mr. Cyril H. Walker.

### THIRTY-THIRD LIST OF BOOKS.

The titles of books mentioned in previous lists are not repeated.

The figures in brackets refer to the figures after the names of the donors, and show by whom the volumes were presented. The books to which no such figures are attached have either been bought from the Library Fund or received through the *Journal*.

**Allbutt, T. C.** [Ed.] *A System of Medicine*. Vol. VII. ... ... ... ... 1899
**Anderson, J. W.** *The Power of Nature in Disease* ... ... ... ... ... 1899
**Anstie, F. E.** ... *Notes on Epidemics* ... ... ... ... ... ... ... ... (1) [1866]
**Armitage, T. R.** ... *Hydropathy as applied to Acute Disease* ... ... ... (1) 1852
**Baden, G. L.** ... *Lægevidenskabens Historie* ... ... .. ... ... (4) 1823
**Bauke, A. C.** ... *Medical Officer* ... ... ... ... ... ... ... ... (1) 1860
**Bell, B.** ... ... *A System of Surgery* (1) Vols. I., New Ed., 1790, II., 3rd Ed., 1787, III., 4th Ed., 1789, IV., 3rd Ed. 1789
**Blundell, J.** ... *Vorlesungen über Geburtshilfe* (Deutsch bearbeitet von L. Calmann). 2. Hauptabth. ... ... (4) 1838
**Boeck, W.** ... ... *Recherches sur la Syphilis* ... ... ... ... ... (4) 1862
**Bonorden, H. F.** ... *Die Syphilis* ... .. ... ... ... ... ... ... (4) 1834
**Briggs, F. H. and H. F.** *Advanced Scientific Dentistry* ... ... ... (1) [N.D.]
**Brindel, [—]** ... *Des Lésions de la Table interne du Crane dans les Suppurations de l'Oreille moyenne* ... ... ... ... 1899
**Brodie, [G. J.] Guthrie und [B. C.]** *Vorlesungen über die vorzüglichsten Krankheiten der Harnausführungsorgane und des Mastdarms* (Deutsch bearbeitet des F. J. Behrend) ... ... ... ... ... ... ... (4) 1836
**Browne, L.** ... ... *The Throat and Nose, and their Diseases* .. 5th Ed. 1899
**Bruel, W.** ... ... *Praxis Medicinæ, or, The Physicians Practise* (1) 2nd Ed. 1639
**Burns, J.** ... ... *Handbuch der Geburtshülfe* (Herausgegeben von H. F. Kilian) ... ... ... ... ... ... ... (4) 1834
*Buxton: its History, Waters, Climate, Scenery, etc.* ... ... ... ... ... [N.D.]
*Carlsbad, Guide to* ... ... ... ... ... ... ... ... ... ... (1) [1886]
**Carus, C. G.** ... *Lehrbuch der Gynäkologie*. 2 vols. ... ... ... (4) 1820
**Clutterbuck, J. Wardrop und H.** *Blutentziehungen in Krankheiten* (Deutsch bearbeitet des F. J. Behrend) ... (4) 1840
**Coblentz, V.** .. *The Newer Remedies* ... ... ... ... ... 3rd Ed. 1899
*Creuznach-Spa and its Environs* ... ... ... ... ... ... ... ... 1899
**Dick, R.** ... ... *Diet and Regimen* ... ... ... ... ... (1) 2nd Ed. 1839
**Dover, T.** ... ... *The Ancient Physician's Legacy to his Country* (1) 8th Ed. 1771
**Druitt, R.** ... ... *Haandbog i Chirurgien* (Oversat af A. Arndtsen) (4) 1856
**[Dutt, W. A.]** ... *Lowestoft as a Winter Resort* ... ... ... ... ... ... 1899
**Elder and J. S. Fowler, G.** *The Diseases of Children* ... ... ... ... 1899
**Ellis, H.** ... ... *Mescal: A New Artificial Paradise* ... .. ... ... 1898
*Encyklopädie der gesammten Medicin.* (Herausgegeben von C. C. Schmidt) (4) 6 bd. 1841-42, Supplementbd. I., 1843, II.—IV. 2. Aufl. 1849
**Erichsen, J. E.** ... *Praktisches Handbuch der Chirurgie* (Übersetzt von O. Thamhayn). 2 vols. ... ... ... ... (4) 1864
**Fitzgerald, J.** ... *Pyorrhœa Alveolaris* ... ... ... ... ... ... ... [1899]

**Foster, M.** ... ... *Recent Progress in Physiology* ... ... ... ... ... 1898

**Fowler, G. Elder and J. S.** *The Diseases of Children* ... ... ... ... ... 1899

**Fry, D. P.** ... ... *Law of Vaccination* ... ... ... ... ... (1) 5th Ed. 1872

**Gant, F. J.** ... ... *Guide to the Examinations by the Conjoint Examining Board in England.* 7th Ed. (Ed. by W. Evans) 1899

**Goodhart, J. F.** ... *The Diseases of Children* ... ... .. ... ... 6th Ed. 1899

**Gräfe, E. A.** ... *Neues pratisches Formulare und Recepttaschenbuch* (4) 1834

**Grafstrom, A. V.** *Medical Gymnastics* ... ... ... ... ... ... ... ... ... 1899

**Gregory, J.** ... ... *Conspectus Medicinæ Theoreticæ.* (Translated) (1) [2nd Ed.] 1833

**Guthrie und [B. C.] Brodie, [G. J.]** *Vorlesungen über die vorzüglichsten Krankheiten der Harnausführungsorgane und des Mastdarms* (Deutsch bearbeitet des F. J. Behrend) ... ... .. ... ... ... ... ... (4) 1836

**Harrison, R.** ... *Stone, Prostate and other Urinary Disorders* ... ... 1899

**Herschell, G.** ... *Constipation* ... ... ... ... ... ... .. 2nd Ed. 1899

**Hewer, Mrs. L.** ... *Our Baby* ... ... ... ... ... ... ... ... 6th Ed. 1899

**Hooper, R.** ... ... *Medical Dictionary* ... ... ... ... ... (1) [wants title]

**Hudson-Cox and J. Stokes, F.** *The Pocket Pharmacopœia* ... ... .. ... 1899

*Isle of Man, The—Official Guide* .. ... ... ... ... ... ... ... (2) 1899

**Johnson, G.** ... *On Epidemic Diarrhœa and Cholera* ... ... ... (1) 1866

**Kingscote, E.** ... *Asthma* ... ... ... ... ... ... ... ... ... .. ... 1899

**Külz, E.** ... ... *Klinische Enfahrungen über Diabetes mellitus* ... ... 1899

**Landerer, A.** ... *Treatment of Tuberculosis with Cinnamic Acid.* (Translated) ... ... ... ... ... ... ... ... ... ... [N.D.]

**Latham, P. M.** ... *Diagnostik durch das Gehör bei Krankheiten der Brust* (Deutsch bearbeitet des F. J. Behrend) ... (4) 1837

*Lexicon of Medicine and the Allied Sciences* (New Syd. Soc.). Vol. V. ... 1899

**Liebig, J.** ... ... *Chemistry in its Application to Agriculture and Physiology.* 2nd Ed. (Ed. by L. Playfair) ... (1) 1842

**Lisfranc, [J.]** ... *Krankheiten des Uterus* (Deutsch bearbeitet des F. J. Behrend) ... ... ... ... ... ... ... ... (4) 1839

**Lockwood, C. B.** ... *Aseptic Surgery* ... ... ... ... ... ... ... 2nd Ed. 1899

**Lumley, W. G.** ... *The Medical Officer's Manual* ... ... ... ... (1) 3rd Ed. 1871

**M., G. E. F.** ... *Medical Missions* ... ... ... ... ... ... ... ... ... [N.D.]

**McCaw, J.** ... ... *Aids to the Diagnosis and Treatment of Diseases of Children (Medical)* .. ... ... ... ... ... 2nd Ed. 1899

**Mac Donald, A.** ... *Experimental Study of Children* ... ... ... ... ... 1899

**Macilwain, G.** ... *On the Unity of the Body* ... ... ... ... ... (1) 1836

**Mackintosh, D. J.** *Skiagraphic Atlas of Fractures and Dislocations* ... ... 1899

**Magendie, F.** ... *Vorlesungen über organische Physik* (Deutsch bearbeitet des F. J. Behrend) ... ... ... ... (4) 1836

,, ... *Vorlesungen über organische Physik* (Uebersetzt von G. Krupp) Band III. ... ... ... ... ... ... (4) 183

,, ... *Vorlesungen über das Blut* (Aus dem Französischen von G. Krupp) ... ... ... ... ... ... ... (4) 1839

**Moor, T. H. Pearmain and C. G.** *Aids to the Analysis of Food and Drugs* 2nd Ed. [1899]

**Morellus, P.** ... *The Expert Doctor's Dispensatory.* (Translated) (1) 1657

**Morison, A.** ... *The Nervous System and Visceral Disease* ... ... ... 1899

**Moullin, C. M.** ... *Enlargement of the Prostate* ... ... ... ... 2nd Ed. 1899
*Nederlandsch Gasthuis voor Ooglijders, Utrecht, De Voltooiing van het* ... (9) 1899
**Niemeyer, F. v.** ... *Lehrbuch der speciellen Pathologie und Therapie.* 2 vols. ... ... ... ... ... ... (4) 7th Ed. 1868
*Nurses, The Pocket Case Book for* ... ... ... ... ... ... ... ... ... ... [N.D.]
**Parham, F. W.** ... *Thoracic Resection for Tumors growing from the Bony Wall of the Chest* ... ... ... ... ... ... ... 1899
**Pearmain and C. G. Moor, T. H.** *Aids to the Analysis of Food and Drugs* 2nd Ed. [1899]
**Petit, [C.-A.]** ... *Sur la Cure à Royat* ... ... ... ... ... ... ... 1899
**Plenck, J. J.** ... *Novum Systema Tumorum* ... ... ... ... ... (1) 1767
**Quinn, J. H.** ... *Manual of Library Cataloguing* ... ... ... ... ... 1899
**Rieder, H.** ... ... *Atlas of Urinary Sediments.* (Tr. by F. C. Moore. Ed. by A. S. Delépine) .. ... ... ... ... 1899
**Shaw, J.** ... ... *Golden Rules of Psychiatry* ... ... ... ... ... .. [1899]
**Siebold, E. C. J. von.** *Lehrbuch der Geburtshülfe* ... ... ... (4) 2nd Ed. 1854
**Smith, H.** ... .. *Hæmorrhoids and Prolapsus of the Rectum* (1) 3rd Ed. 1862
,, ... ... *The Surgery of the Rectum* ... ... ... ... ... (1) 1865
**Squire, P.** ... ... *Companion to the British Pharmacopœia.* 17th Ed. (Revised by P. W. Squire) ... ... ... ... ... 1899
**Stokes, F. Hudson-Cox and J.** *The Pocket Pharmacopœia* ... ... ... ... 1899
**Taylor, J. W.** ... *Extra-Uterine Pregnancy* ... ... ... ... ... ... 1899
**Thompson, Sir H.** *Urinorganernes Sygdomme* (Pa Dansk ved J. Blicher) (4) 1871
,, *Modern Cremation* ... ... ... ... ... ... 3rd Ed. 1899
**Thomson, StC.** ... *The Cerebro-Spinal Fluid* ... ... ... ... ... ... 1899
**Treves, F.** ... ... *Intestinal Obstruction* ... ... ... ... [2nd Ed.] 1899
*Typhoid Fever in the Metropolis of Sydney, N.S.W., The Sanitary Value of the Operations of the Board in Reducing and Avoiding the Mortality from* ... ... ... ... [1898]
*Vegetable Physiology and Botany* ... ... ... ... ... ... ... ... ... (1) 1842
**Venables, R.** ... *Elements of Urinary Analysis and Diagnosis* ... (1) [1850]
**Walker, N.** ... ... *An Introduction to Dermatology* ... ... ... ... ... ... 1899
**Walshe, W. H.** ... *Lungernes Sygdomme* (Oversat af J. Blicher) ... (4) 1861
**Wardrop und H. Clutterbuck, J.** *Blutentziehungen in Krankheiten* (Deutsch bearbeitet des F. J. Behrend) ... (4) 1840
**West, C.** ... ... *Fremstilling af Bornesygdommenes* (Bearbeitet af F. V. Mansa) ... ... ... ... ... ... ... (4) 1855
,, ... ... *Fremstilling af Qvindens Sygdomme* (Bearbeitet af F. V. Mansa) ... ... ... ... ... ... ... (4) 1860
**Wilson, E. M.** ... *Notes on Malaria in Connection with Meteorological Conditions at Sierra Leone.* 3rd Year ... ... ... 1899
*Xeroform* ... ... ... ... ... ... ... ... ... ... ... ... ... ... [N.D.]
*Ziekenverpleging en de zorg voor de openbare gezondheid in de laatste 50 jaren, De* 1899

---

### TRANSACTIONS, REPORTS, JOURNALS, &c.

American Association of Obstetricians and Gynecologists, Transactions of the ... ... ... ... ... ... Vols. IX.—XI. 1897-99
American Gynæcological and Obstetrical Journal, The .. Vol. XIV. 1899

American Journal of Obstetrics, The ... Vol. XXXIX. 1899
American Journal of the Medical Sciences, The ... Vol. CXVII. 1899
American Laryngological Association, Transactions of the ... 1899
American Public Health Association, Reports and Papers of the Vol. XXIV. 1898
Archives cliniques de Bordeaux ... Tome VII. 1898
Archives of Clinical Skiagraphy ... [Vol. I.] 1896
Archives of Pediatrics, The ... Vol. XV. 1898
Archives of the Roentgen Ray ... Vol. II. 1898
Boston Medical and Surgical Journal, The Vols. CXXXIX., CXL. 1898–99
Bookseller, The ... (1) 1884–98
Bristol Port Sanitary District—Annual Report for 1898 ... 1899
British Gynæcological Journal, The ... Vol. XIV. 1898
British Medical Journal, The ... Vol. I. for 1899
Bulletin de l'Institut international de Bibliographie ... 1897
China Medical Missionary Journal, The ... Vols. XI., XII. 1897–98
Cincinnati Lancet-Clinic, The ... Vol. LXXXI. 1899
Dermatological Society of Great Britain and Ireland, Transactions of the ... Vol. IV. 1898
Dublin Journal of Medical Science, The ... Vol. CVII. 1899
Edinburgh Medical Journal, The ... N.S., Vol. V. 1899
Gazette de Gynécologie ... Tome XIII. 1898
Giornale internazionale delle Scienze mediche ... 1898
Glasgow Medical Journal, The ... Vol. LI. 1899
Gloucester Court Guide and County Blue Book, The ... 1899
Grant College Medical Society, Transactions of the ... 1899
Hospital, The ... Vol. XXV. 1899
Hospitals—
Glasgow Hospital Reports ... Vol. I. 1898
King's College Hospital Reports ... Vol. V. 1899
Indian Medical Gazette, The ... Vol. XXXIII. 1898
International Directory of Laryngologists and Otologists ... 1899
International Medical Congress, 1881.—Catalogue of Temporary Museum ... (1) 1881
Johns Hopkins Hospital Bulletin, The ... Vol. IX. 1898
Journal de Neurologie ... Tomes II., III. 1897–98
Journal of Experimental Medicine, The ... Vol. III. 1898
Journal of Nervous and Mental Disease, The ... Vol. XXV. 1898
Journal of the American Medical Association, Vol. XXXII., 1899; General Index to Vols. I.—XXIV. ... 1896
Journal of the Sanitary Institute ... Vol. XIX. 1898
Lancet, The ... Vol. I. for 1899
Library Association Year Book, The ... 1899
Library World, The ... Vol. I. 1899
London, Report on the Sanitary Condition of the City of, for 1898 (7) 1899
McGill University, Faculty of Medicine, Calendar of the ... (3) 1899
Medical and Surgical "Review of Reviews," The ... Vol. I. 1898
Medical News, The ... Vols. LXXIII., LXXIV. 1898–99
Medical Press and Circular, The ... [Vol. CXVIII.] 1899
Medical Record ... Vol. LV. 1899

| | | |
|---|---|---|
| Monthly Cyclopædia of Practical Medicine, The | Vol. I. | 1898 |
| Münchener medicinische Wochenschrift | | 1898 |
| New York Medical Journal, The | Vol. LXIX. | 1899 |
| Obstetrical Transactions | Vol. XL. | 1899 |
| Philadelphia Medical Journal, The | Vol. III. | 1899 |
| Practitioner, The | Vol. LXII. | 1899 |
| Railway Surgeon, The | (5) Vol. V. | 1898–99 |
| Retrospect of Medicine, Braithwaite's | Vol. CXIX. | 1899 |
| Royal Medical and Chirurgical Society of London, Proceedings of the | Vols. I.—IX., 1857–82; N.S., Vol. I., 1885; (6) 3rd Ser., III., IV., 1891–92, VI., VII., 1894–95, IX. | 1898 |
| St. Petersburger medicinische Wochenschrift | | 1898 |
| Sanitary Record, The | Vol. XXIII. | 1899 |
| Scottish Medical and Surgical Journal, The | Vol. IV. | 1899 |
| Virginia Medical Semi-Monthly | (8) Vol. III. | 1899 |
| Year-Book of Scientific and Learned Societies | | 1899 |

### Local Medical Notes.

### BARNSTAPLE.

Public Health.—Dr. Mark Jackson has been re-appointed Medical Officer of Health. Dr. R. Gooding has been appointed Medical Officer of Health for the Barnstaple Port Sanitary District, *vice* Mr. E. Rouse, deceased.

Vaccination Returns.—At a meeting of the Board of Guardians held on July 21st, the Chairman remarked that vaccination was on the increase in their union; in one district £9 were paid for vaccination fees during the past quarter, whereas in 1898 only £15 were paid for the whole year.

### BARRY.

New Nursing Home.—With the rapid growth of Barry, the work of the nursing staff has steadily increased, and the New Victoria Jubilee Nurses' Home, which has just been completed, was very badly needed. It is a handsome and commodious structure, and has been erected entirely by means of voluntary subscriptions.

### BATH.

Public Health.—Dr. W. H. Symons has been appointed Medical Officer of Health for the City and County of Bath. At a meeting of the Town Council it was decided to increase the salary of the medical officer from £200 to £435 per annum.

Prevention of Consumption.—At a meeting of the Sanitary Committee on June 26th the Medical Officer of Health (Dr. Symons) presented a report, in which he stated that there were last year 59 deaths from phthisis, and from other tuberculous diseases 28. In 1868 the deaths from phthisis in Bath numbered 132, and from other tuberculous diseases 70. This saving from death and from illness was probably due to sanitary and social improvement. Their death-rate from phthisis is now less than that for England and Wales; but they ought not to be satisfied as long as any persons died of phthisis. It had been decided to form a branch of the National Association for the Prevention of Consumption for the Counties of Gloucestershire, Somerset, and Wilts. Other large towns were falling in with the movement, and Dr. Symons hoped Bath would not be behindhand. So far, he thought, they were doing more than was being done in many places. They disinfected after deaths from phthisis wherever they could get permission to do so, and they always visited the houses and left circulars as to the means to be adopted to avoid the disease. They had not yet got regulations under the Dairies, Cowsheds, and Milk-shops Order, but these were now under consideration. As to the practical results likely to follow the new movement, it appeared to him that they might teach persons suffering from phthisis how to carry out successful treatment at home by giving them a few weeks' treatment in a public institution where every detail was under medical supervision. Recent results showed that phthisis could be cured as well in England as elsewhere.

Royal United Hospital.—Mr. Frederick C. Forster has been appointed House Surgeon.

Eastern Dispensary.—Mr. D. Leslie Beath has been appointed Honorary Medical Officer.

Obituary.—The death is reported of Mr. John Terry. The deceased qualified as M.R.C.S. in 1844, and L.S.A. in 1845. Mr. Terry was a very old member of the Bath and Bristol Branch of the British Medical Association, and for many years served on the Council. He was a constant attendant at the meetings, and took an active part in discussions relating to mental diseases, in which, as proprietor of Bailbrook Asylum, he was most interested.

### BRENTRY.

Home for Inebriates.—At the meeting of the Bristol Town Council held on June 14th, it was decided to make a conditional grant of £2,000 to the Royal Victoria Home for Inebriates at Brentry. The Cardiff Watch Committee, at their meeting on June 14th, decided to accept the offer of the authorities of the Inebriates' Home at Brentry, and to send patients there at a cost of £10 a year each, with 3s. 6d. per day for maintenance, such provision to be for not less than five inmates and for five years.

### BRISTOL.

Ham Green Fever Hospital.—On the invitation of the Health Committee, a large number of medical men visited the new Corporation Fever Hospital at Ham Green, on July 14th. As a pamphlet containing full information on the structure of the pavilions aud other buildings was presented to each present, there is no need to go into details ; it will suffice to say that the wards are a model of what a hospital should be, the bed-space being more than ample, the sanitary arrangements and conveniences perfect, and every possible care taken to render the place suitable for the reception of sick people. Of the surroundings nothing could have appeared on that day more charming, the beautiful lawns, magnificent cedars and other trees, and the charming views made the visitors almost wish they were convalescents, and compelled to stop there for some weeks at the city's expense. The latest report is that some patients are already there, but, as the Medical Officer of Health said, the less the place is used the better he will be pleased, as it will indicate an absence of infectious diseases in the city. We cannot conclude without congratulating Mr. Yabbicom, Dr. Davies, and the Hospital Sub-committee on the satisfactory completion of their labours, for they have supplied Bristol with a Fever Hospital second to none in the kingdom.

Infirmary Nurses' Home.—The Carnival at the Zoological Gardens held to raise enough money to furnish the Nurses' Home at the Royal Infirmary was one of the most, if not the most, successful entertainment of that sort ever held in Bristol, and what is more gratifying, everybody was pleased, and more than sufficient money was received, though we have no doubt some worthy object will be found at that Institution for the surplus. Firstly, the weather was all that it should be, the gardens were at their best, and the willing band of ladies who took stalls or helped in the refreshment places, the medical students and many gentlemen who helped at the shooting galleries and Aunt Sally booths threw all their energies into "the cause," and extracted the not unwilling pence from the spectators. The bicycle gymkhana was very entertaining and some wonderful riding was shown by a company of ladies and gentlemen from Weston. Lastly, but by no means leastly, the maypole dance by small children was very pretty, and elicited hearty applause at each performance.

### CARDIFF.

Public Health.—A deputation from the Cardiff Medical Society has informed the Health Committee of the Cardiff Corporation that the members of the Society were prepared to notify to the Medical Officers of Health cases of tuberculosis coming under their notice, with a view to infected rooms and articles being disinfected by the Authority. No fee will be paid by the Corporation for the information.

The Public Health Laboratory, which was established last year by the Glamorgan County Council, will in future be under the joint charge of the Medical Officers of Health of Cardiff and of the county, under the name of the "Cardiff and County Public Health Laboratory." A bacteriological laboratory has been erected and fitted up by the County Council at a cost of about £500, and the current expenses of the institution will be shared during the next two years by the two Authorities. A bacteriologist is to be appointed at a salary of £300 per annum, and he is to have a qualified assistant. It is expected that arrangements will be made with the authorities of University College whereby the bacteriologist appointed may become a Professor of the College and give lectures on hygienic subjects.

Infirmary.—Dr. W. M. Stevens has been appointed Pathologist.

### CHEPSTOW.

Public Health.—Mr. G. Lawrence has been appointed Medical Officer of Health, *vice* Mr. E. P. King resigned.

### CHELTENHAM.

Hospital for Sick Children.—Mr. C. E. Lansdown has been appointed Surgeon.

### CHIPPING SODBURY.

Public Health.—Mr. A. D. Parr-Dudley has been appointed Medical Officer for the Fifth Sanitary District.

Vaccination Literature.—At a meeting of the Chipping Sodbury Board of Guardians a letter was read from Dr. F. T. Bond, Honorary Secretary of the Jenner Society, enclosing specimen tracts and leaflets on the subject of vaccination. One of the members thought they should also have some of the large posters issued by the Society placed in two villages where information was much needed upon the matter. It was decided to purchase some large posters and copies of several of the tracts and leaflets.

### DEVONPORT.

Royal Albert Hospital.—At a meeting of the Subscribers of this Institution it was decided to erect a new Nurses' Home at a cost of £2,200, and to provide isolation wards, consulting rooms for the medical staff, etc., at an estimated expenditure of about £1,500.

Infirmary.—The Infirmary Committee of the Devonport Board of Guardians, in an exhaustive report on the nursing staff and the management and general condition of the infirmary, suggest that the Local Government Board should be urged to issue an order directing that matrons of workhouses should have no jurisdiction in infirmaries, but that the superintendent nurse should exercise paramount authority.

Memorial to the late Mr. L. P. Metham.—At the annual meeting of the Royal Female Orphan Asylum it was decided to perpetuate the memory of the late Mr. L. P. Metham, M.R.C.S.Eng., L.S.A., who had been Honorary Secretary to the institution from its foundation, by raising a fund, to be called the "Metham Endowment Fund," for the maintenance of five beds to be filled by children nominated from time to time by his two daughters.

Honorary Freemanship to a Medical Man.—On July 25th the honorary freemanship of the borough of Devonport was presented to Alderman Joseph May, F.R.C.S.Eng., L.S.A., J.P. The certificate presented to Mr. May contained sketches of the municipal and other public buildings, made allusion to the "eminent services rendered to the borough" by him, and recorded his municipal appointments as follows: Commissioner, 1840-94; councillor, 1861-63; alderman, 1868-99; mayor, 1870-71, 1871-72, 1872-73, 1875-76. The casket was of silver-gilt, richly embellished and suitably inscribed.

### EXETER.

Devon and Exeter Hospital.—T.R.H. the Duke and Duchess of York visited the Devon and Exeter Hospital on July 4th. Afterwards a garden party was held in the grounds, and 120 little girls presented the Duchess with purses of £5 each, on behalf of the hospital.

### GLOUCESTER.

Public Health.—In his annual report, Dr. Bond, the Medical Officer of Health to the Gloucestershire Combined Sanitary District, states that the birth-rate was 26.4 per 1,000 of the estimated population. The general birth-rate has varied during the last ten years beween 29.6 and 25.9, but with a distinct tendency to decline, as might be expected in a district which is so largely agricultural in its character. The total number of deaths registered during the year was 1,264, being a diminution of 137, or 9.7, on the mortality of the previous year, and giving an average death-rate of 12.9 for the whole district, the best for the last quarter of a century.

### HELSTON.

Vaccination.—At the meeting of the Board of Guardians held on July 22nd, it was reported that the vaccination expenses for the last quarter amounted to £107, being £72 in excess of the preceding quarter. The Clerk stated that at present the cost of vaccination would average about £180 yearly, while under the old system it never exceeded £50 per annum. The Guardians decided to petition the Local Government Board to reduce the minimum fees to be paid to Public Vaccinators.

### ILFRACOMBE.

Public Health.—Dr. E. Slade-King has submitted to the Devon County Council his annual abstract of the reports made by the Medical Officers of Health at the close of the year 1898, relative to the several rural and urban districts of North Devon, together with his comments. He says: "The standard reached by the majority of the reports is excellent, and is the outcome of earnest, practical work, conducted on the lines of skilful sanitary training. The vital statistics for North Devon are: Birth-rate, 24; death-rate, 14.5; zymotic death-rate, .9; infant death-rate per 1,000 births, 112.5; notification of infectious disease per 1,000 population, 3.2. The vital statistics for England and Wales are: Birth-rate, 29.4; death-rate, 17.6; zymotic death-rate, 2.22; infant mortality per 1,000 births, 161."

### KEYNSHAM.

Sewerage Scheme.—The Keynsham Rural District Council has received a letter from the Bristol Sanitary Authority to the effect that the Sanitary Committee of the Council of Bristol was preparing a scheme to divert the sewage of Bristol from the river Avon, and to discharge it into the Bristol Channel at Avonmouth. The proposed outfall sewer of the Corporation could easily be made available for the use of the inhabitants of all the areas draining into the rivers Avon and Frome, up to and including Bath; and it appeared to the Corporation that drainage by means of such a sewer would be more efficient and less costly than by systems of sewage treatment adopted independently by the several Local Authorities. The Keynsham Rural District Council was invited to discuss terms with the Bristol Corporation for the use of the proposed sewer. The Keynsham Council decided that this invitation should be accepted.

Tuberculosis.—The Keynsham Board of Guardians, at a recent meeting, supported a resolution from the St. Saviour's Union, Surrey, urging the Local Government Board to make an exhaustive inquiry into the causes and treatment of tuberculosis.

### MILLBROOK.

Memorial to the late Dr. A. B. Cheves.—Some friends have placed a beautiful stained-glass window in the side chapel of the parish church as a memorial to the late Dr. Alexander Bruce Cheves.

### NEWPORT.

Water-works.—The expenditure on the Wentwood Water-works up to the present has been £160,000, and it is estimated that the works will not be completed for another two and a half years. The original estimate was £120,000, and the contract was let for £93,000, but the Newport Corporation subsequently undertook the work themselves.

### NEWQUAY.

Public Health.—The report of the Medical Officer of Health indicates that sanitary matters are well attended to at this popular seaside resort. The death-rate for 1898 was 14.7. A small epidemic of scarlet fever was promptly and efficiently dealt with, and the isolation hospital proved of great value in limiting the outbreak. In the Meteorological Report we notice the equable character of the temperature; and the fact that the lowest recorded temperature was 32.9° shows the mildness of the climate.

### NEWTON ABBOT.

Vaccination.—At the meeting of the Board of Guardians held on July 12th, the Vaccination Committee reported that the new Act had cost the Guardians during the past half-year £168 more than the old Act in the corresponding half of last year. The public vaccinators received £192 for 591 cases, as against £45 for 437 cases in the corresponding period of the preceding twelve months; the average cost of each case being 6s. 6d. against 2s. 1d. of last year. The Chairman of the Committee stated that the medical officers were dissatisfied with their fees, and he thought that the Committee would be compelled to recommend the Guardians to increase the fees from 6s. to 7s. 6d. per case.

### PENARTH.

Diphtheria.—The District Council have decided to convert the upper portion of the Council offices into a temporary hospital for the isolation of cases of diphtheria.

### PENZANCE.

Public Health.—Mr. R. D. Boase has been appointed Medical Officer by the Port Sanitary Authority.

### REDRUTH.

West Cornwall Women's Hospital.—The children's ward recently added was formally opened by Mr. Passmore Edwards on May 17th. The ward is to commemorate the Queen's Diamond Jubilee.

### SALISBURY.

Infirmary.—Dr. Edmund T. Fyson has been appointed Physician.

### STRATTON.

Public Health.—Mr. S. H. Rentzsch has been appointed Medical Officer of Health for the South District of Stratton Union.

### SWANSEA.

General Hospital.—The annual meeting was held on July 13th. The report for 1898 showed 1,307 in-patients had been admitted during the year. The average length of stay of each was thirty days, and the daily number of occupied beds was eighty-four. 4,636 out-patients had been treated. The financial statement showed that the ordinary income amounted to £4,761, being an increase of £272 as compared with 1897. The ordinary expenditure was £4,906, a decrease of £118 with the previous year. It was decided to spend £1,000 in extending the heating arrangements of the hospital. On May 18th the Mayor of Swansea (Mr. R. Martin) opened the Victoria Nurses' Home, erected in connection with the Swansea General Hospital, at a cost of £1,800, in commemoration of the Queen's Diamond Jubilee.

### THORNBURY.

Vaccination Fees.—At a meeting of the Thornbury Board of Guardians, held on May 26th, a communication was read from two of the medical officers, asking that the vaccination fees might be on a higher scale than those fixed by the Local Government Board. The majority of the Guardians were of opinion that they had acted fairly to the medical officers by fixing the fess above the minimum laid down by the Act, and as these officers had appealed to the Local Government Board, it was decided that the fees should, therefore, remain the same as suggested by the Local Government Board.

### TIVERTON.

Public Health.—Mr. J. R. R. Pollock has been appointed Medical Officer of Health.

Sewerage Scheme.—A Local Government Board inquiry was recently held at Tiverton into the Town Council's application for sanction to borrow £6,750 for purposes of sewerage and sewage disposal. Mr. J. Siddalls described the scheme of sewerage. The district (West Axe) to be sewered contained a population of about 2,800. As the district was low-lying, it had been found necessary to divide it into two portions.

Infirmary.—The recent bazaar held in aid of the Tiverton Infirmary Building Fund, after paying all expenses, realised nearly £600.

### TORQUAY.

Sewerage Scheme. — Recently Major General Carey, Chief Engineering Inspector of the Local Government Board, presided at a Conference between Torquay Town Council and St. Marychurch District Council relative to the discharge of additional sewage by St. Marychurch into the Torquay sewers. Mr. F. S. Hex, Town Clerk, acted on behalf of Torquay Town Council, and Mr. Grant Wollen for St. Marychurch. Both Councils were strongly represented. St. Marychurch Council had prepared a new drainage scheme that will carry additional sewage into the Torquay main, and the Torquay Council protested.

### TOTNES.

Cottage Hospital.—Drs. J. L. Cuppaidge, G. J. Gibson, Mr. J. L. C. Hains, and Dr. K. R. Smith have been re-appointed Medical Officers.

### TROWBRIDGE.

Prevention of Tuberculosis.—A small but influential meeting, under the auspices of the West Wiltshire Branch of the National Association for the Prevention of Consumption and other Forms of Tuberculosis was held on July 29th. Captain Chaloner, M.P., presided, and was supported by the Bishop of Salisbury, Dr. E. Markham Skerritt, Dr. F. R. Walters, and others. Dr. Markham Skerritt, alluding to a special report on tuberculosis presented to the Health Committee of the Bristol Town Council by Dr. Davies, said that during the last ten years the seven principal zymotic diseases caused 4,673 deaths in Bristol, while 3,520 were credited to pulmonary consumption alone, and the deaths from tuberculosis as a whole amounted to 4,876 in the same period, or more than those for the zymotic diseases put together. One death in every nine was due to some form of tuberculosis, and one in every twelve to consumption of the lungs. The Bishop of Salisbury also spoke, suggesting the Wiltshire Downs as an admirable site for a sanatorium. On the motion of Mr. C. Awdry, seconded by Sir John Wallington, it was agreed to amalgamate Gloucestershire, Somersetshire, and Wiltshire, for the purpose of making a joint effort against tuberculosis.

### TRURO.

Public Health.—Mr. C. P. Mathew has been appointed Medical Officer for the Tregony Sanitary District.

Tuberculosis.—At the last meeting of the Cornwall County Council it was resolved :—"That the objects of the Devon and Cornwall Branch of the National Association for the Prevention of Consumption are deserving such support as the County Council can give, and, pending the further consideration of the matter, we recommend the Council to bear the cost of publishing throughout the county information with regard to these diseases and their prevention."

### WARMLEY.

Public Health.—Mr. F. W. S. Stone has been appointed Medical Officer for the Bitton Sanitary Distict.

Vaccination Returns.—At a meeting of the Board of Guardians held on July 18th, it was reported that the number of successful vaccinations for the half-year ending June 30th, 1898, was 106, and for the first six months of 1899 it was nearly double that amount—viz., 208.

### WELLS.

Public Health.—Dr. C. G. MacVicker has been appointed Medical Officer and Public Vaccinator for the Fourth Sanitary District, *vice* Dr. F. J. Malden resigned.

